# PReS13-SPK-1137: New developments in our care & understanding of JIA

**DOI:** 10.1186/1546-0096-11-S2-I7

**Published:** 2013-12-05

**Authors:** MW Beresford

**Affiliations:** 1 Institute of Translational Medicine, University of Liverpool, Liverpool, UK

## 

Despite significant advances in our care and understanding of patients with Juvenile Idiopathic Arthritis (JIA), many continue to have severe, debilitating disease. A holistic multi-disciplinary approach to the care of children and their families is critical to maximise the child's physical and psychosocial development. Landmark clinical trials including more recently those of a range of biologics have significantly improved outcomes. These include trials of tocilizumab, canakinumab, and other ongoing trials that are the result of important international collaborative efforts. However predicting which children will respond in a timely manner remains an evolving art as children may be exposed to potentially toxic agents whilst finding the most suited for them. Long-term safety, as well as efficacy, is critically important.

Knowledge translation that arises in the day to day care of children into driving forward ever greater understanding of the biological basis that underpins JIA and its sub-types is ever more crucial. Similarly, our understanding of the underlying biological processes and our ability to manipulate the immune pathways with ever more accuracy offer enormous clinical opportunities, yet also challenges. Involvement of the muti-disciplinary team will ensure that best care is provided going forward.

An overview of recent advances in our care and understanding of children with JIA will focus on recent and novel treatments that are now being introduced into our care of patients.

## Disclosure of interest

None declared.

